# Biochemical and Genetic Characterization of Ergot Alkaloid Biosynthesis in *Aspergillus aspearensis*

**DOI:** 10.3390/toxins18010047

**Published:** 2026-01-16

**Authors:** Jessica L. Fuss, Daniel G. Panaccione

**Affiliations:** School of Natural Resources and the Environment, West Virginia University, Morgantown, WV 26506, USA; jlf00024@mix.wvu.edu

**Keywords:** ergot alkaloids, *Aspergillus*, lysergic acid amides, biosynthetic gene clusters, entomopathogenesis

## Abstract

Ergot alkaloids derived from lysergic acid have impacted humankind significantly as toxins in agriculture and as the foundations of several pharmaceuticals. Few fungi capable of producing lysergic acid derivatives have been found outside the family Clavicipitaceae. Based on its phylogenetic placement, we hypothesized the recently described fungus *Aspergillus aspearensis* (Aspergillaceae) would synthesize lysergic acid amides. Cultures of *A. aspearensis* produced abundant lysergic acid α-hydroxyethylamide (LAH) and lesser amounts of other lysergic acid derivatives. Conidia contained high concentrations of ergot alkaloids, whereas sclerotia contained significantly less. Approximately half of the ergot alkaloids produced were secreted into the culture medium. When spores of *A. aspearensis* were injected into larvae of the model insect *Galleria mellonella*, larvae died at a significantly faster rate than control larvae. The fungus produced ergot alkaloids during insect pathogenesis and later produced conidia and sclerotia on cadavers, indicating it can complete its life cycle in an insect. The genome of *A. aspearensis* contained two complete ergot alkaloid synthesis gene clusters, similar to those of *A. leporis*; however, unlike its sister species, none of the ergot cluster genes were pseudogenized. *Aspergillus aspearensis* is a newly discovered source of ergot alkaloids and may be useful for studying and producing these important chemicals.

## 1. Introduction

Ergot alkaloids produced by fungi are significant in agriculture and medicine in multiple ways. Ways in which ergot alkaloids affect agriculture include the historical poisoning of people through ingestion of ergot sclerotia of *Claviceps purpurea* that contaminated infected grain crops. Consumption of ergot alkaloids found in high concentrations in these fungal structures led to a condition called ergotism that resulted in significant human suffering and death in certain cultures through much of recorded history [1,2]. Similar ergot alkaloids produced by *Epichloë* species that are endophytic symbionts of grasses continue to plague animal agriculture by reducing animal growth, health, and reproduction [3,4]. Conversely, the anti-herbivore activities of ergot alkaloids reduce damage by insect pests of grasses associated with *Epichloë* symbionts [5,6] and also may be factors in the success of related *Metarhizium* species as insect biocontrol agents [7,8]. In medicine, ergot alkaloids have been the foundations of numerous pharmaceuticals prescribed to treat dementia, migraines, Parkinson’s disease, and a variety of other conditions [9,10,11]. The potent pharmacology of ergot alkaloids is exemplified in the activities of the semi-synthetic ergot alkaloid derivative LSD [12]. For these reasons, knowledge of the distribution of ergot alkaloid biosynthetic capability in fungi of different lineages and occupying different ecological niches is important.

**Figure 1:**
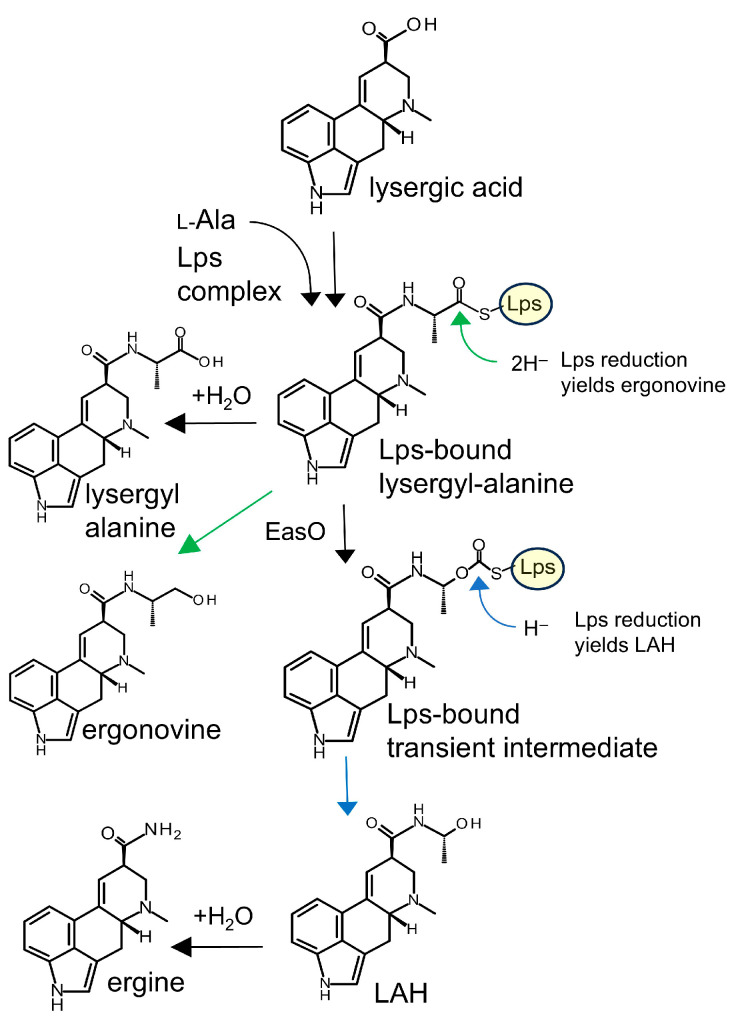
Pathway from lysergic acid to lysergic acid amides observed in Aspergillus species. Lps: lysergyl peptide synthetase; LAH, lysergic acid α-hydroxyethylamide. Green arrows indicate reduction to ergonovine, whereas blue arrows indicate reduction to LAH.

Different classes of ergot alkaloids are produced from alternate branches of a highly complex pathway [10,11]. The class of ergot alkaloids most closely associated with the toxicological and pharmaceutical properties described above comprises the lysergic acid-derivatives. The *Claviceps*, *Epichloë*, and *Metarhizium* species discussed above are all members of the family Clavicipitaceae and all produce lysergic acid-derived ergot alkaloids from a shared pathway encoded by genes comprising a biosynthetic gene cluster referred to as an *eas* cluster [11,13]. Outside of fungi in the Clavicipitaceae, the ability to produce lysergic acid-derived ergot alkaloids has only been documented in four fungi representing two additional, diverse orders of fungi. These include three species of *Aspergillus* (order Eurotiales) [14] and a distantly related species of *Dicyma* (order Xylariales) [15]. Molecular and bioinformatic analyses of the three *Aspergillus* species revealed independent evolution of some pathway steps and a gene with novel biosynthetic capacity [14]. No genetic data are available for the *Dicyma* species. The *Aspergillus* species accumulate the lysergic acid amides ergonovine and lysergic acid α-hydroxyethylamide (LAH), along with hydrolysis products lysergyl-alanine and ergine (Figure 1), with LAH typically being produced in the greatest abundance.

The three *Aspergillus* species documented to produce lysergic acid-derived ergot alkaloids include: *A. leporis*, which was originally isolated from rabbit dung in Wyoming [16], a western US state, and also has been found in the rhizospheres of plants in that same geographic region [17]; *A. hancockii* from agricultural soils and commodities in Australia [18]; and, *A. homomorphus* from soil near the Dead Sea of Israel [19] as well as on millet in Yemen [20]. *Aspergillus leporis* and *A. hancockii* are close relatives in *Aspergillus* section *Flavi*, whereas *A. homomorphus* is in a separate *Aspergillus* section, *Nigri*. A noteworthy difference in the *eas* clusters of the *Aspergillus* species compared to those of the Clavicipitaceae is the inclusion of a single, two-module peptide synthetase-encoding gene in the *Aspergillus* species as opposed to two or three separate types of peptide synthetase genes whose products must work in different combinations to make lysergic acid amides (such as ergonovine and LAH) or ergopeptines in the Clavicipitaceae [11,21]. Phylogenetic analyses indicated that the ability to produce lysergic acid evolved once in an ancestor to both the *Aspergillus* species and the members of the Clavicipitaceae, but the ability to assemble derivatives of lysergic acid evolved independently in the two lineages [14].

Recently, a new species of *Aspergillus*, *A. aspearensis* Houbraken, Frisvad, Arzanlou & Samson, was described after being isolated from soil from Aspear Island in Urmia Lake of Iran [22]. Based on phylogenetic analyses of a concatenated dataset derived from genes encoding β-tubulin, calmodulin, and the second largest subunit of RNA polymerase II, *A. aspearensis* grouped as a sister species to *A. leporis* in a clade within section *Flavi* that contained *A. leporis* and *A. hancockii* as its only other members [22]. Considering the phylogenetic placement of this new species, we hypothesized that *A. aspearensis* would produce ergot alkaloids and tested that hypothesis by chemical and genomic approaches.

## 2. Results

### 2.1. Accumulation of Lysergic Acid Amides in Cultures of A. aspearensis

**Figure 2:**
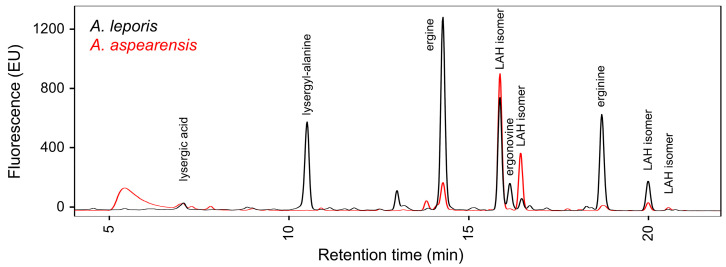
Fluorescent analytes from *A. aspearensis* elute at the same times as ergot alkaloids of *A. leporis* in high-performance liquid chromatography. Analytes were detected by fluorescence with excitation at 310 nm and emission at 410 nm. EU: emission units; LAH, lysergic acid α-hydroxyethylamide. Ergot alkaloids derived from lysergic acid stereoisomerize in protic solvents, often leading to detectable stereoisomers; LAH has two stereocenters, yielding four potential stereoisomers.

Cultures of *A. aspearensis* isolate CBS 143673 grown on malt extract agar for two weeks at 30 °C were analyzed for the presence of ergot alkaloids. Extracts were derived from 400-μL samples containing all fungal material present in that region of the culture dish (hyphae, conidia, and, potentially, immature sclerotia) as well as the agar medium (to the bottom of the Petri dish) on which the sampled portion of the culture had grown. Extracts of *A. aspearensis* contained abundant ergot alkaloids of the lysergic acid amide class—most abundantly the stereoisomers of LAH and its hydrolysis product, ergine—as assessed by comparison to extracts of *A. leporis* in high-performance liquid chromatography (HPLC) analyses (Figure 2). Analytes from the *A. leporis* extracts had been characterized previously as the specified lysergic acid derivatives by additional instrumental approaches [14].

Ten other culture media were assessed for their ability to support LAH accumulation in *A. aspearensis* in a survey conducted with individual cultures. Among media tested, only pyrithiamine medium [23] and acetamide medium [24] supported accumulation of LAH to concentrations similar to those obtained on malt extract agar (Table S1). All other tested media resulted in accumulation of LAH to concentrations less than 20% of that obtained on malt extract agar. Malt extract agar, as opposed to either of the other high-yielding media, was chosen for conducting all other culture-based experiments because of its low cost and its support of production of abundant conidia and sclerotia.

**Figure 3:**
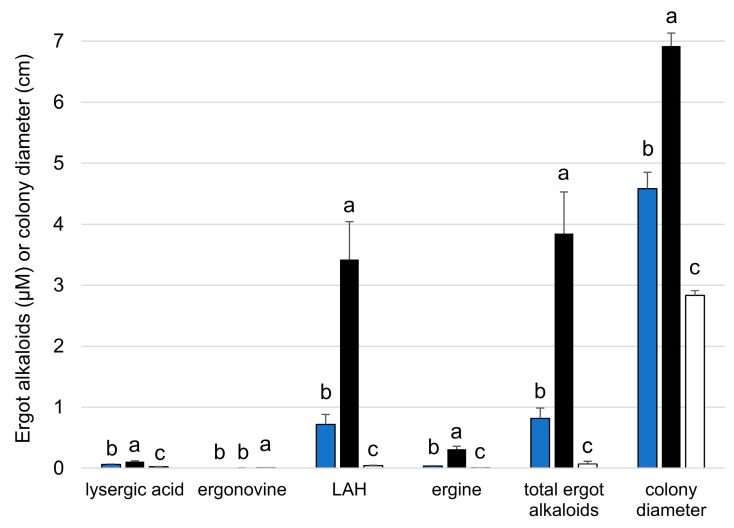
Accumulation of ergot alkaloids in cultures of *A. aspearensis* grown on malt extract agar for 12 days at different temperatures. Values represent mean ± standard error for eight cultures at each temperature. Values within each alkaloid type or colony diameter represented by bars marked with different letters differed significantly in a nonparametric Steel–Dwass multiple comparison test (*p* < 0.05). Ergot alkaloid concentrations were calculated based on a standard curve of ergonovine and thus, with the exception of ergonovine, must be considered ‘relative to ergonovine’ as opposed to absolute. Lysergyl-alanine was not detectable in these 12-day cultures of *A. aspearensis*.

Quantitative analyses of ergot alkaloids and the effect of temperature on ergot alkaloid accumulation and colony diameter were investigated in cultures grown on malt extract agar for 12 days. Cultures grown at 30 °C had significantly greater colony diameter and significantly greater concentrations of LAH and other ergot alkaloids with the exception of ergonovine, which was not a significant component of the ergot alkaloid profile at any temperature (Figure 3). The increase in ergot alkaloid accumulation at 30 °C suggests that either ergot alkaloid synthesis gene expression or enzyme activity is greatest at that temperature. Our data do not allow us to discriminate between these two possibilities.

Since ergot alkaloids were detected in complex samples of *A. aspearensis* containing hyphae, conidia, sclerotia (in some cases), and the agar medium on which the fungus had grown, we tried to separate some of these components to better assess where ergot alkaloids were accumulating. Conidia harvested separately from the remainder of their source culture contained abundant ergot alkaloids, whereas sclerotia separated from their source culture contained notably lower yields of ergot alkaloids per g dry weight than observed in conidia (Table 1). During the preparation of conidial extracts we weighed dried samples containing enumerated conidia, allowing us to calculate the average mass of an individual conidium of *A. aspearensis* as 4.5 ± 1.0 pg (mean ± standard error). That mass is intermediate between the masses calculated previously for conidia of *A. fumigatus* (2.9 ± 0.3 pg) and *A. nidulans* (8.8 ± 1.8 pg) [25].

To separate culture medium from the fungus, static test tube cultures were grown in 500 μL of malt extract broth. In the static liquid cultures, 46% of the total ergot alkaloids were secreted into the culture medium with the remaining 54% associated with the solid phase (hyphae, conidia, and perhaps immature sclerotia) (Table 2). Lysergic acid and ergonovine, which were minor contributors (in terms of quantity) to the ergot alkaloid profile, appeared to be secreted at higher rates than LAH or ergine. In a statistical test, however, ergot alkaloid species only marginally affected secretion in a one-way ANOVA (*p* = 0.06). Overall, the data indicate that about half of the total ergot alkaloids of *A. aspearensis* are secreted and that the half remaining in the solid phase is distributed mainly between hyphae and conidia (with very little in sclerotia). Our experimental approach did not allow us to cleanly assign separate alkaloid concentration values to hyphae, because we could not obtain hyphal samples that were free of conidia.

### 2.2. Infection of Galleria mellonella Larvae by A. aspearensis Is Associated with Lethality and the Accumulation of Lysergic Acid Amides

**Figure 4:**
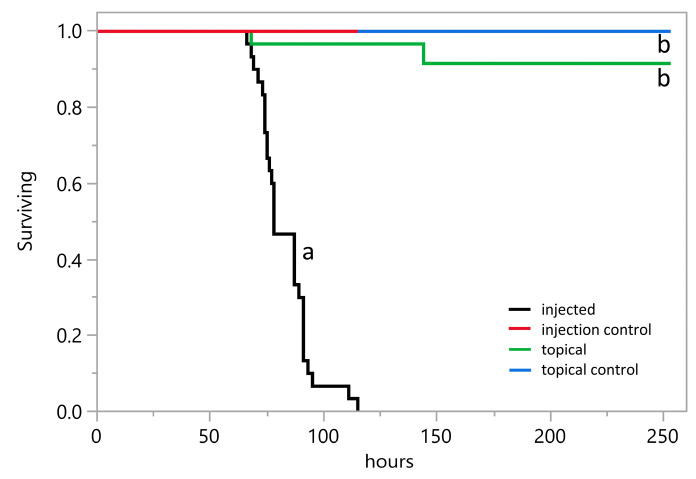
Survival of larvae of *G. mellonella* after inoculation with conidia of *A. aspearensis* by injection (20 μL at 3000 conidia/μL) or topically (exposure to conidiating culture) compared to control larvae not inoculated with fungus. Lines marked with different lower-case letters indicate survival rates that differed significantly in a log rank test with Bonferroni-adjusted alpha set at 0.0083.

Because the related fungus *A. leporis* parasitized insects and produced ergot alkaloids in insects [26], we tested the pathogenic potential of *A. aspearensis* in similar assays. Injection of conidia of *A. aspearensis* into larvae of the model lepidopteran *G. mellonella* resulted in death of all inoculated larvae over 3 to 4 days with an LT_50_ (median time to lethality) over three trials of 80 ± 6 hr (mean ± standard deviation) (Figure 4). The LT_50_ of *A. aspearensis* was similar to that observed with *A. leporis* (85 ± 10 hr) in previous trials conducted with similar concentrations of conidial inoculum [26]. No mortality was observed over this same time period in larvae that had been mock inoculated with a control solution of phosphate-buffered saline (PBS). The difference in survival curves for *A. aspearensis*-inoculated larvae and control larvae was statistically significant in a log rank test (*p* < 0.0001, with Bonferroni-adjusted alpha set at 0.0083).

**Figure 5:**
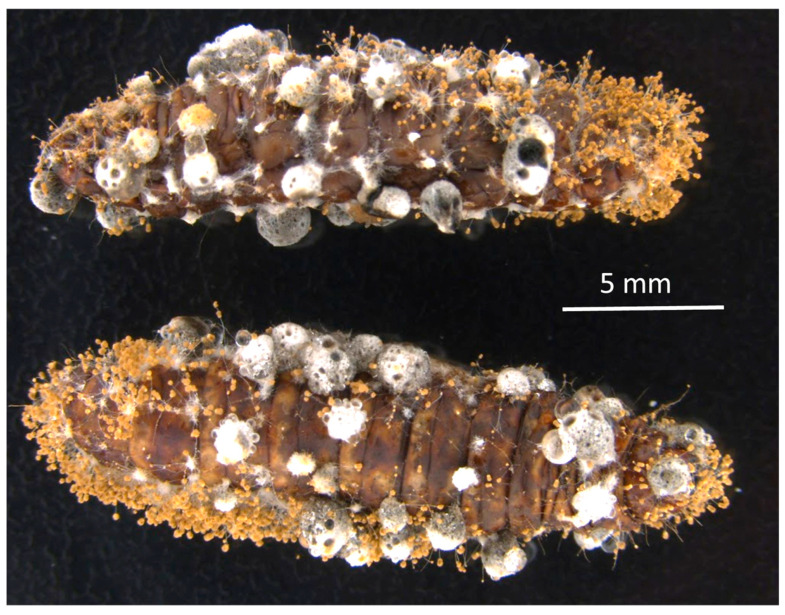
Larvae of *G. mellonella* bearing conidia and sclerotia of *A. aspearensis*.

*Aspergillus aspearensis* emerged from several dead larvae and conidiated and formed sclerotia on cadavers (Figure 5), indicating that the fungus can complete its asexual life cycle on an insect host. When larvae of *G. mellonella* were inoculated topically with *A. aspearensis* by placing them on sporulating cultures, only 2 of 30 larvae (among three trials) died over a subsequent 10-day observation period (Figure 4). The survival curves for larvae topically inoculated with *A. aspearensis* compared to non-inoculated controls did not differ significantly in a log rank test (*p* = 0.15, with Bonferroni-adjusted alpha set at 0.0083). These data suggest that although *A. aspearensis* has significant pathogenic potential, it lacks an effective mechanism to penetrate the insect’s cuticle. In the absence of such a mechanism, the fungus would need to infect through wounds or be vectored by some agent capable of cuticle penetration. Effective insect pathogens rely on appressoria and/or cuticle-degrading enzymes to penetrate an insect hosts’ cuticle [7].

**Figure 6:**
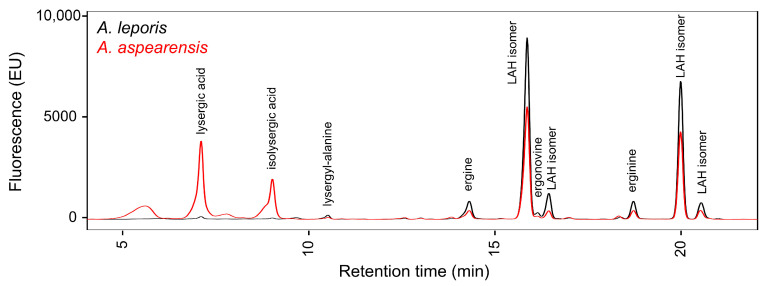
HPLC analyses of ergot alkaloids extracted from larvae of *G. mellonella* injected with *A. aspearensis* or *A. leporis*. Alkaloids were extracted 12 days after inoculation.

**Figure 7:**
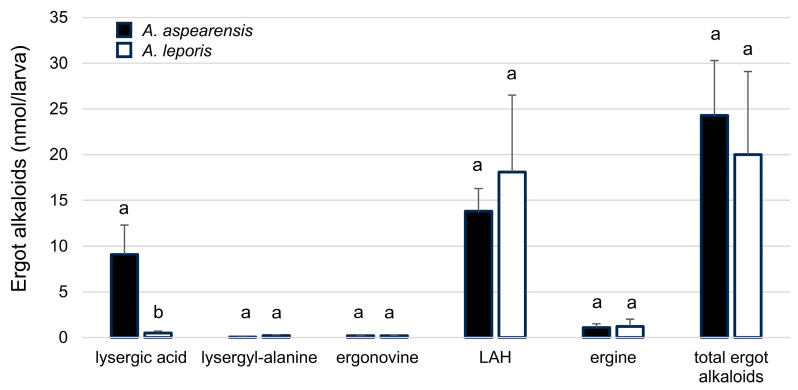
Accumulation of ergot alkaloids in larvae of *G. mellonella* injected with *A. aspearensis* or *A. leporis*. Alkaloids were analyzed 12 days after inoculation. Values within each alkaloid type represented by bars marked with different letters differed significantly in ANOVA (*p* < 0.05). Concentrations were based on a standard curve of ergonovine and thus, with the exception of ergonovine, must be considered ‘relative to ergonovine’ as opposed to absolute.

Ergot alkaloids accumulated in insect cadavers consumed by *A. aspearensis*. The same lysergic acid amides detected in cultures of *A. aspearensis* (and *A. leporis*) were observed in infected larvae (Figure 6). The concentrations of most ergot alkaloids that accumulated in *G. mellonella* infected with *A. aspearensis* and *A. leporis* were similar (Figure 7). One exception was a significantly higher concentration of lysergic acid in the larvae infected with *A. aspearensis*. Previous studies have demonstrated the significance of LAH to insect pathogenesis by *A. leporis* [26] and *M. brunneum* [8]. Any potential contribution of lysergic acid to insect pathogenesis has not been tested and should not be inferred from these results.

### 2.3. Ergot Alkaloid Synthesis Genes in A. aspearensis

**Figure 8:**
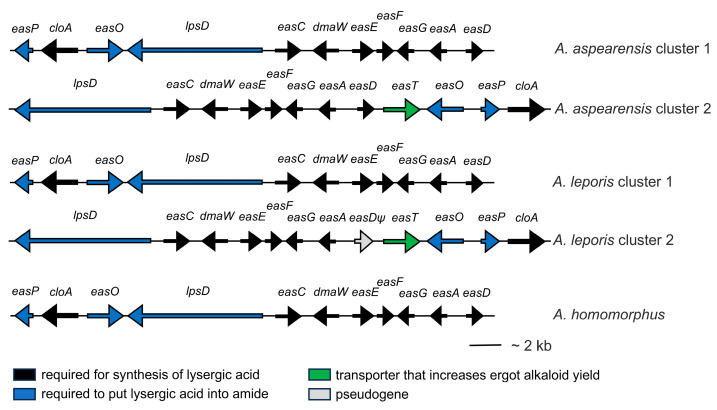
The two ergot alkaloid synthesis (*eas*) clusters of *A. aspearensis*, compared with the two *eas* clusters of *A. leporis* and the sole *eas* cluster of *A. homomorphus.* Genes required for synthesizing lysergic acid are shown in black, whereas those involved in putting lysergic acid into lysergic acid amides are shown in blue. The gene shown in green (*easT*) encodes a major facilitator superfamily transporter that increases ergot alkaloid yield but is not required for ergot alkaloid synthesis [27]. The gray arrow for *easD_ψ_* in *A. leporis* cluster 2 is so marked because it is a pseudogene [14]. Representations of data from *A. leporis* and *A. homomorphus* are used with permission of the American Society for Microbiology.

A draft genome assembly of Illumina technology-based reads of *A. aspearensis* CBS 143673 yielded a genome of 41.3 Mb over 2085 contigs, with 77-fold coverage, an N_50_ of 396 kb, and an L_50_ of 26. The genome contained two separate contigs with ergot alkaloid synthesis (*eas*) gene clusters that each contained all the necessary genes to encode the biosynthesis of LAH (Figure 8). One contig (GenBank accession JBIGLF010000097) contained an ergot alkaloid synthesis (*eas*) cluster that was similar to, and syntenic with, *eas* cluster 1 of *A. leporis* and the lone *eas* cluster of *Aspergillus homomorphus* [14]. The second contig (GenBank accession JBIGLF010000037) contained an *eas* cluster that was syntenic with *eas* cluster 2 of *A. leporis* [14], including a homolog of the major facilitator superfamily transporter gene, *easT* [27], which had until now not been identified in other *eas* clusters. One noteworthy difference between *A. aspearensis eas* cluster 2 and *eas* cluster 2 of *A. leporis*, is that the gene *easD*, which is a pseudogene in *A. leporis eas* cluster 2, has no frame shifts or premature stop codons in the second *eas* cluster of *A. aspearensis* and thus appears to encode a functioning protein.

In addition to the two LAH-associated gene clusters, the previously studied genome of *A. leporis* is unusual in having two satellite clusters that encode some of the genes required to make products of two different ergot alkaloid pathway branches: fumigaclavine A and rugulovasines A/B [28]. In order to be functional, these satellite clusters in *A. leporis* must rely on activities of some of the core *eas* genes encoded in the LAH-associated clusters in *A. leporis*. Based on the similarity of *A. aspearensis* to *A. leporis*, we searched the genome of *A. aspearensis* for evidence of full or partial biosynthetic gene clusters for these alternate ergot alkaloid pathway products. Blastn searches with *easH* or *easQ* from the rugulovasine satellite cluster of *A. leporis* identified a contig (GenBank accession JBIGLF010000052) containing homologs of *easH* and *easQ* adjacent to each other and oriented to be transcribed divergently as they are in *A. leporis* [28]. We obtained no evidence of accumulation of rugulovasines in *A. aspearensis*. Conditions under which rugulovasines are produced in *A. leporis* are not well defined; in multiple analyses in *A. leporis* only a single culture was observed to contain the stereoisomeric pair rugulovasine A and B [28]. Sequences flanking *easH* and *easQ* in *A. aspearensis* were explored by blastx searches but no additional ergot alkaloid synthesis genes were discovered within 20 kb adjacent on either side of the *easH* and *easQ* mini-cluster. Unlike the genome of *A. leporis*, no cluster, complete or partial, capable of contributing to fumigaclavine A synthesis was detected in the genome of *A. aspearensis*. Critical genes in this branch for which there were no reasonable homologs in *A. aspearensis* included reductase allele of *easA*, and the fumigaclavine A branch-associated genes *easM* and *easN*. There were no convincing homologs for ergot alkaloid synthesis genes in the genome of *A. aspearensis* outside of the clusters detailed above.

## 3. Discussion

The production of lysergic acid amides by *A. aspearensis* is a rare property among free-living, soil-inhabiting fungi. *Aspergillus aspearensis* shares this exceptional ability with three other soil-inhabiting *Aspergillus* species from different geographic regions: *A. leporis* (from the western USA), *A. hancockii* (Australia), and *A. homomorphus* (Israel and Yemen) [14]. Our results also add to the noteworthy association of lysergic acid amides—LAH in particular—with soil inhabiting fungi that have the potential to parasitize insects. In addition to the *Aspergillus* species, several *Metarhizium* species produce LAH (most prominently among other lysergic acid amides) and are insect-infecting, rhizosphere inhabitants [7,29]. In another example, *Periglandula clandestina*, though it symbiotically inhabits the shoots of morning glories, sends much of its lysergic acid amides to the roots of its host plant [30] where they may contribute to resistance to biotic stresses [31,32]. By contrast the other major class of lysergic acid derivatives, ergopeptines, has been documented mainly from plant shoot or seed inhabiting organisms such as *Epichloë* and *Claviceps* species and exert their effects on organisms that interact with those above-ground tissues [2,5,6].

The profile of ergot alkaloids observed in cultures *A. aspearensis* was qualitatively similar to that observed in *A. leporis*, though fungal cultures were grown on different media (as was necessary to get significant yields of ergot alkaloids from the different species). Cultures of *A. aspearensis* retained much of their ergot alkaloids in LAH, whereas *A. leporis* tended to have larger shares of lysergyl-alanine and ergine—hydrolysis products derived from an intermediate step and from LAH, respectively—than did *A. aspearensis* (Figure 2). In insects, where it was possible to directly compare quantities of ergot alkaloids of the two species on the same substrate, both species contained mainly LAH but *A. aspearensis* accumulated significantly more lysergic acid than did *A. leporis* in this context.

Our analyses of ergot alkaloid accumulation in cultures of *A. aspearensis* revealed that the lysergic acid amides accumulated in more than one part of the culture. Approximately half of the ergot alkaloid yield was secreted into the culture medium, and the other half was retained in the solid phase. Jones et al. [14] reported 66% of the ergot alkaloid content of *A. leporis* was secreted, a value similar to what we measured in *A. aspearensis*. Conidia of *A. aspearensis* contained relatively high concentrations of lysergic acid amides: 3.2 µmol/g dry weight. This concentration is on the same order of magnitude with the 8.1 µmol/g dry weight value observed for fumigaclavines—products of a different branch of the ergot alkaloid pathway—in *A. fumigatus*, a fungus renowned for concentrating its ergot alkaloids in its conidia [33]. Sclerotia of *A. aspearensis*, however, contained very little of the lysergic acid amides: 7.7 nmol/g dry weight (almost 500-fold lower per unit weight than in conidia). This observation is somewhat surprising because sclerotia are the primary site of accumulation of ergot alkaloids in ergot fungi (*Claviceps* species), where typical concentrations of ergot alkaloids have been documented to be approximately one-thousand-fold higher (in the 1 to 10 µmol/g range) in *C. purpurea* and *C. africana* [34,35] than in *A. aspearensis* sclerotia. Sclerotia are stress resisting structures and may contain alkaloids to protect them from biotic stresses. Frisvad et al. [22] noted accumulation of several other classes of specialized metabolites (an aflavinine, kojic acid, mevinolins, and paspalinines) in *A. aspearensis*, raising the possibility that these fungal structures may be protected by alternate classes of chemicals.

*Aspergillus aspearensis* demonstrated good pathogenic potential in the model insect *G. mellonella*, killing insects at a rate comparable to that previously established with *A. leporis* [26]. *Aspergillus aspearensis* was only an effective pathogen, however, when injected into the hemocoel, suggesting that it does not have a good mechanism to breach the insect cuticle. In previous experiments [26] insects topically inoculated with *A. leporis* died significantly faster than uninoculated control larvae; though injection was still a more effective inoculation method for *A. leporis*. The production of conidia and sclerotia of *A. aspearensis* on dead insects was striking and indicated that *A. aspearensis* can complete its life cycle on an insect. Moreover, the ergot alkaloids of *A. aspearensis* accumulated to very high concentrations—approximately 24 nmol per larva. Considering an approximate volume of 200 µL per larva, the measured quantity of ergot alkaloids translates to approximately 100 µM. This insect-associated concentration is approximately 25-fold higher than the concentration observed in malt extract cultures of *A. aspearensis* of about the same age. An increase in lysergic acid amide concentration in insects relative to cultures also has been documented in *Metarhizium* species [29].

The genome of *A. aspearensis* contained two complete biosynthetic gene clusters, each of which had the capacity to encode the biosynthesis of LAH. The presence of the lysergic acid amides predicted by the gene sequences shows that at least one of each homologous gene pair is functional; assessment of functionality of genes from both *eas* clusters in this context is theoretical and based on the lack of frame shifts or premature stop codons in their coding sequences. In other sequenced examples in which ergot alkaloid producers had two *eas* clusters, at least one of the duplicated genes was pseudogenized. These examples include pseudogenization of *easD* in *eas* cluster 2 of *A. leporis* [14] and pseudogenization of a copy of *lpsB* in one of two *eas* clusters in *Epichloë coenophiala* [36]. Notably *eas* cluster 1 and *eas* cluster 2 of *A. aspearensis* are not duplicates of each other in terms of gene synteny or even gene content. This point is true for the two clusters of *A. leporis*, as well. Thus, the two *eas* clusters most likely did not arise by a recent duplication event in either of the *Aspergillus* species. Cluster 1 is syntenic with the lone *eas* cluster of *A. homomorphus*, whereas cluster 2 has an additional gene—*easT* encoding a major facilitator superfamily transporter—and has two changes in gene order or gene position relative to *eas* cluster 1. Previously, *easT* was characterized functionally in *A. leporis* and found to contribute to ergot alkaloid synthesis (quantitatively) but not secretion [27]. The pseudogenization of *easD* in cluster 2 of *A. leporis* presumably happened after *A. leporis* and *A. aspearensis* diverged from a recent common ancestor. The lack of pseudogenization of any of the *A. aspearensis eas* genes may be due simply to random probability (since only one gene is pseudogenized in *A. leporis*) or to some unidentified selective pressure that differs between the two fungi due to their ecological niches or geography. Interestingly, *A. hancockii*, which also is part of the *A. leporis* phylogenetic clade [22], contains several pseudogenized *eas* genes in its genome though it maintains at least one functional copy of each essential gene and makes lysergic acid amides [14]. The genome of *A. aspearensis* lacked some of the complexity of ergot alkaloid synthesis genes noted previously in *A. leporis* [28] in that it lacked the partial, satellite gene cluster involved in making fumigaclavine A in *A. leporis*. Differences in specialized metabolites other than ergot alkaloids were noted in previous studies of *A. aspearensis* and *A. leporis* [22]. *Aspergillus leporis*, produced six classes of specialized metabolites that were not present in similarly cultivated *A. aspearensis*; conversely, *A. aspearensis* produced two other classes of specialized metabolites that were not detected in *A. leporis*.

Lysergic acid amides have now been documented in all three members (*A. aspearensis*, *A. leporis*, and *A. hancockii*) of the *A. leporis* clade, a small clade embedded within the large section *Flavi* of the highly speciose genus *Aspergillus* [22]. The ergot alkaloid synthesis gene clusters of these *Aspergillus* species are complex and diverse. Reasons why these particular species harbor these gene clusters and produce these bioactive alkaloids whereas their phylogenetic relatives do not may be a fruitful area for future phylogenetic and chemical ecology-based investigations. Most other producers of lysergic acid-based ergot alkaloids are plant-associated members of the Clavicipitaceae, many of which do not grow well or produce ergot alkaloids well away from their plant hosts. Thus, fast-growing saprotrophic ergot alkaloid producers like *A. aspearensis*, with their wealth of genetic resources, also may have translational significance for research and development relevant to understanding, modifying, controlling, or overexpressing this important group of agricultural and pharmaceutical chemicals.

## 4. Materials and Methods

### 4.1. Fungi and Culture Conditions

*Aspergillus aspearensis* Houbraken, Frisvad, Arzanlou & Samson strain CBS 143673 was obtained from The Westerdijk Fungal Biodiversity Institute, Utrecht, The Netherlands, and maintained on malt extract agar (per liter: 6 g malt extract, 1.8 g maltose, 6 g dextrose, 1.2 g yeast extract, 15 g agar [34]). Unless time or temperature were varied as part of a specific experiment (in which case, details are provided for that experiment), cultures were grown for two weeks at 30 °C in an incubator with ambient oxygen. *Aspergillus aspearensis* was grown on additional media (24 days at room temperature), to test for ergot alkaloid accumulation, including pyrithiamine medium [23], acetamide medium [24], bar maintenance medium [37], coconut agar [38], Rose Bengal agar [39], Czapek-Dox agar [40], *Neurospora* minimal medium [40], sucrose-yeast extract agar [29], Luria–Bertani agar [41], and Gamborg’s medium [42]. *Aspergillus leporis* States & M. Chr. strain NRRL 3216 was obtained from the USDA Agricultural Research Service Culture Collection (NRRL) at Peoria, Illinois, USA, and was cultivated as a source of reference material for lysergic acid amides. *Aspergillus leporis* was grown on sucrose-yeast extract medium [29], which was previously shown to promote excellent accumulation of lysergic acid amides in that fungus [14].

To collect conidia of *A. aspearensis* separately from hyphae and sclerotia, malt extract agar plates containing one-month-old cultures of the fungus were inverted (such that the lid was down) and tapped several times to dislodge conidia so that they would collect on the inner surface of the lid. Any sclerotia that landed among the conidia were removed with forceps. Conidia were then harvested from the inner surface of the lid by washing it with 50% methanol. An aliquot was counted on a hemacytometer after diluting in water containing 0.1% tween 20. Conidia were then collected on a pre-weighed 0.2-µm-pore nylon centrifugal filter and dried in a speed-vac before weighing. Sclerotia were manually collected separately from conidia and hyphae, dried in a speed-vac, and weighed prior to extraction of ergot alkaloids.

To compare accumulation of ergot alkaloids in culture fluids as opposed to the solid phase (hyphae, conidia, and, potentially, immature sclerotia) of the fungus, cultures were grown from 50,000 conidia in 0.5-mL of malt extract medium (lacking agar) contained in a 2-mL screw-cap tubes with the lids unscrewed one-quarter turn to provide aeration. After two weeks at 30 °C, the liquid phase of the culture was removed with a micropipette and the volume was measured. The solid phase of the culture was dried in a speed-vac and its dry weight determined. Ergot alkaloids were extracted from liquid by the addition of an equal volume of methanol and from the solid phases by the additional of 1 mL of methanol and bead beating as described immediately below.

Ergot alkaloids were extracted by bead beating cultured fungi or infected insects in methanol as previously described [8,14,26,29]. Briefly, samples were extracted in 2-mL screw-cap microcentrifuge tubes (capped with lids containing rubber O-rings) containing 10 three-mm glass beads. Samples were pulverized by bead beating at 6 m/s for 30 s in a FastPrep 101 (Bio101, Carlsbad, CA, USA) and clarified by centrifugation prior to analysis.

### 4.2. Inoculation of Insects

To test whether the fungi could grow and produce ergot alkaloids in live insects, conidia (60,000 conidia in 20 μL) in modified phosphate-buffered saline (PBS) [33] were injected into the hindmost proleg of larvae of the greater wax moth, *Galleria mellonella*, according to methods we have used routinely for several species of fungi [8,26,29,33]. Additional larvae were inoculated topically by placing them on a sporulating culture of *A. aspearensis* for 24 h before removing them to an empty Petri dish. Survival of insects inoculated with *A. aspearensis* by either method compared to those inoculated with a sterile PBS solution was monitored and recorded every few hours till all treated larvae were dead or to a maximum of 10 days. All experiments with insects were conducted at room temperature (20 °C to 22 °C). Data were collected from three trials with 10 larvae per treatment per trial.

### 4.3. Analysis of Ergot Alkaloids

HPLC was based on the methods described previously [8,14,29,37] on a Waters (Milford, MA, USA) Arc HPLC system with fluorescence of analytes monitored with excitation at 310 nm and emission at 410 nm. The solid phase was a Prodigy (Phenomenex, Torrance, CA, USA) C_18_ column with the following properties: length of 150 mm, inside diameter of 4.6 mm, and particle diameter of 5 μm. The mobile phase a was multilinear binary gradient combining 5% acetonitrile + 95% 50 mM ammonium acetate (solution A) and 75% acetonitrile + 25% 50 mM ammonium acetate (solution B) at a flow rate of 1 mL/min. The gradient originated at 100% solution A and ramped linearly to 85% solution A + 15% solution B at 8 min, then to 50% solution A/50% solution B at 25 min, and 100% solution B at 30 min. The flow was held at 100% solution B for 5 min before returning to 100% solution A over 5 min and rinsing with 100% solution A for an additional 5 min before the next injection. Identities of peaks containing lysergic acid, lysergyl-alanine, ergine, LAH, and ergonovine were defined by retention times relative to peaks in reference strains that had been previously defined by additional instrumental analyses [8,14,28,29,37]. Ergot alkaloids were quantified by peak areas relative to an external standard curve prepared with commercially available ergonovine (Sigma, St Louis, MO, USA) and thus values (with the exception of values for ergonovine) must be considered as relative to ergonovine as opposed to absolute.

### 4.4. Statistical Analysis of Results

Statistical analyses of ergot alkaloid quantities were conducted as follows. Datasets were first analyzed for unequal variances by Brown-Forsythe tests. Datasets that passed a Brown-Forsythe test (*p* > 0.05) were subsequently analyzed by ANOVA and, when treatment was a significant factor, means were separated in Tukey’s test. Datasets that did not pass a Brown-Forsythe test (*p* < 0.05) were analyzed nonparametrically by a Wilcoxon’s test and, when treatment was a significant factor, a Steel–Dwass multiple comparison test. Survival data from insect pathogenesis experiments were plotted on Kaplan–Meier survival curves and analyzed by log rank tests with a Bonferroni-adjusted alpha set at 0.0083. All statistical analyses were conducted in JMP edition 18 (SAS, Cary, NC, USA).

### 4.5. Genomic Sequence Acquisition and Analysis

The genome of *A. aspearensis* was sequenced by Illumina (San Diego, CA, USA) NextSeq technology with the assistance of the West Virginia University and Marshall University Genomics Core Facilities and assembled at the Marshall University Bioinformatics Core Facility. Raw reads were trimmed in Trimmomatic version 0.39 [43] by application of the following tools of that software package: the ILLUMINACLIP tool was used with default parameters to remove sequencing adapters from the reads, the SLIDINGWINDOW tool was used with a window size of 4 bases and a minimum quality score of 15 to remove low-quality base calls, and finally, the MINLEN tool was used to filter out reads less than 25 bases in length. Trimmed reads were assembled in SPAdes version 3.15.4 [44] with default settings. To estimate genome coverage, we mapped the trimmed reads back to the assembled genome using bbmap version 39.06 [45]. Genome sizes, N50, and L50 statistics were computed in Python version 3.10.13 by parsing the fasta files. The genome has been deposited at GenBank under accession number JBIGLF000000000.

## Figures and Tables

**Table 1:** Accumulation of ergot alkaloids (nmol/g; mean ± standard error) ^1^ in sclerotia (*n* = 8) or conidia (*n* = 6) of *A. aspearensis* grown on malt extract agar for at least 1 month to obtain sufficient sclerotia for analysis.

Tissue	Lysergic Acid	Ergonovine	LAH	Ergine	Total
sclerotia	1.1 ± 0.3	0.1 ± 0.03	4.1 ± 1.6	2.4 ± 0.6	7.7 ± 2.2
conidia	85 ± 38	13 ± 5	2381 ± 610	728 ± 228	3206 ± 854

^1^ Concentrations were based on a standard curve of ergonovine and thus, with the exception of ergonovine, must be considered ‘relative to ergonovine’ as opposed to absolute.

**Table 2:** Accumulation of ergot alkaloids (pmol/culture tube; mean ± standard error; *n* = 3) ^1^ in solid phase versus liquid phase of cultures of *A. aspearensis* grown in 0.5 mL of malt extract broth for 15 days at 30 °C and percent of alkaloid secreted into liquid phase.

Culture Phase/Secreted	Lysergic Acid	Ergonovine	LAH	Ergine	Total
Solid (pmol/tube)	3 ± 0.3	0.5 ± 0.5	129 ± 12	8 ± 1	140 ± 13
Liquid (pmol/tube)	13 ± 6	6 ± 5	104 ± 19	3 ± 1	127 ± 27
Percent secreted	72 ± 14	81 ± 19	44 ± 2	30 ± 7	46 ± 3

^1^ Concentrations were based on a standard curve of ergonovine and thus, except for ergonovine, must be considered ‘relative to ergonovine’ as opposed to absolute.

## Data Availability

The original contributions presented in this study are included in the article and Supplementary Material. Further inquiries can be directed to the corresponding author.

## Supplementary Materials

The following supporting information can be downloaded at: https://www.mdpi.com/article/10.3390/toxins18010047/s1, Table S1: Accumulation of lysergic acid α-hydroxyethylamide (LAH) (µM) in individual cultures of *Aspergillus aspearensis* cultivated for 24 days on the indicated media at 20 °C [23,24,29,33,37,38,39,40,41,42]; File S1: Data used to create Figure 3, Figure 4 and Figure 7 and Table 1 and Table 2 compiled in a Microsoft Excel workbook with separate, labeled tabs.

## Abbreviations

The following abbreviations are used in this manuscript:

LAH	Lysergic acid α-hydroxyethylamide
HPLC	High performance liquid chromatography
PBS	Phosphate-buffered saline
Lps	Lysergyl peptide synthetase
*eas*	Ergot alkaloid synthesis (as applied to genes or gene clusters)
*clo*	Clavine oxidase
*dmaW*	Dimethylallyl-tryptophan synthase
ANOVA	Analysis of variance
