# Supplementary material for: Biochemical and Genetic Characterization of Ergot Alkaloid Biosynthesis in Aspergillus aspearensis
Source: Toxins (Basel). 2026 Jan 16;18(1):47. doi: 10.3390/toxins18010047 (PMC12846141; doi:10.3390/toxins18010047)
Supplement: Supplementary file 1 [file toxins-18-00047-s001.zip › toxins-4087364-Table S1.pdf]

Supplementary Materials: Biochemical and genetic characterization of ergot alkaloid biosynthesis in *Aspergillus aspearensis*

Jessica L. Fuss and Daniel G. Panaccione*

Table S1. Accumulation of lysergic acid α-hydroxyethylamide (LAH) (μM) in individual cultures of Aspergillus aspearensis cultivated for 24 days on the indicated media at 20 °C.

Medium [reference]	LAH
Pyrithiamine medium [23]	0.48
Acetamide medium [24]	0.46
Malt extract agar [33]	0.45
Bar maintenance medium [37]	0.08
Coconut agar [38]	0.05
Rose Bengal agar [39]	0.03
Czapek-Dox agar [40]	0.01
Neurospora minimal medium [40]	<0.01
Sucrose-yeast extract agar [29]	<0.01
Luria-Bertani agar [41]	<0.01
Gamborg's medium [42]	none detected
